# Cervical Rhabdomyosarcoma: A Case Report

**DOI:** 10.7759/cureus.77902

**Published:** 2025-01-24

**Authors:** Israa AlMulai, Asma Fahad, Zuhdi Nagshabandi

**Affiliations:** 1 Obstetrics and Gynaecology, Latifa Hospital, Dubai Health, Dubai, ARE

**Keywords:** cervical polyp, cervical rhabdomyosarcoma, heavy menstrual bleeding, hysterectomy, rhabdomyosarcoma

## Abstract

Rhabdomyosarcomas are rare in adults, and primary cervical rhabdomyosarcomas are even rarer. Due to its rarity, literature on the presentation and management of cervical rhabdomyosarcoma is scarce. We present the case of a 35-year-old woman who presented with abnormal uterine bleeding, one of the most common complaints in gynecology outpatient clinics. Following evaluation and investigations, she was diagnosed with cervical rhabdomyosarcoma. Timely diagnosis and appropriate treatment ensured a good prognosis for the patient. This case report underlines the importance of histopathology in attaining the appropriate diagnosis, which can be lifesaving.

## Introduction

Immature cells programmed to develop into striated skeletal muscle can cause rhabdomyosarcoma. The condition is the most frequent soft tissue sarcoma in children and adolescents, making up almost half of all soft tissue sarcomas and 5% of all pediatric malignancies [1]. However, rhabdomyosarcomas are uncommon in adults, accounting for fewer than 1% of all cancers and fewer than 5% of all soft tissue sarcomas [2]. Primary cervical rhabdomyosarcoma is even rarer. The uterine cervix is one of the least common sites for rhabdomyosarcoma in the genitourinary tract. This tumor comprises 0.2% of the malignant tumors of the uterus affecting adult females [3].

During a 40-year period, Ferguson et al. [4] at Memorial Sloan-Kettering Cancer Center discovered just eight cases of adult cervical rhabdomyosarcoma. In a study performed by Kriseman et al. [5] over a 30-year period, only 11 cases of cervical rhabdomyosarcoma were diagnosed. A study by Devins et al. [6], which included 94 patients with a median age of 23 years, showed a varied clinical presentation, with vaginal bleeding in the majority of the patients, protruding vaginal mass in 17 patients, cervical polyps in eight patients, or expelled tumor fragments per vagina in five cases.

The current management of cervical embryonal rhabdomyosarcoma depends primarily on the patient’s age. As it is more common in younger females, conservative management to retain the genitourinary organs is the preferred modality. Other factors taken into account include the histologic subtype, size, site of origin, disease extent at presentation, and residual disease after treatment [7].

Here, we present the case of a 35-year-old woman who presented with abnormal uterine bleeding, a common presentation in gynecology outpatient clinics. Evaluation and histopathology resulted in a timely diagnosis of a rare presentation of cervical rhabdomyosarcoma which led to prompt treatment and ensured a good prognosis for the patient. This presentation is similar to one of the two cases reported by de Kock et al. [8], where a 44-year-old woman presented with a history of abnormal uterine bleeding, and the examination showed a cervical polyp that was subsequently diagnosed as cervical embryonal rhabdomyosarcoma.

Due to its rarity, there is a paucity of data on cervical rhabdomyosarcoma. This case report will add to the available literature aiding clinicians to recognize this unusual lesion and have a high index of suspicion in similar cases.

## Case presentation

A 35-year-old woman from Uganda presented to our gynecology outpatient clinic with a complaint of abnormal uterine bleeding for the past year. She complained of heavy menstrual periods since menarche at age 13, which increased over the past year before the presentation. No pap smear was performed before the presentation, and there was no family history of cancer. The patient had a history of three normal vaginal deliveries and no miscarriages.

**Figure 1:**
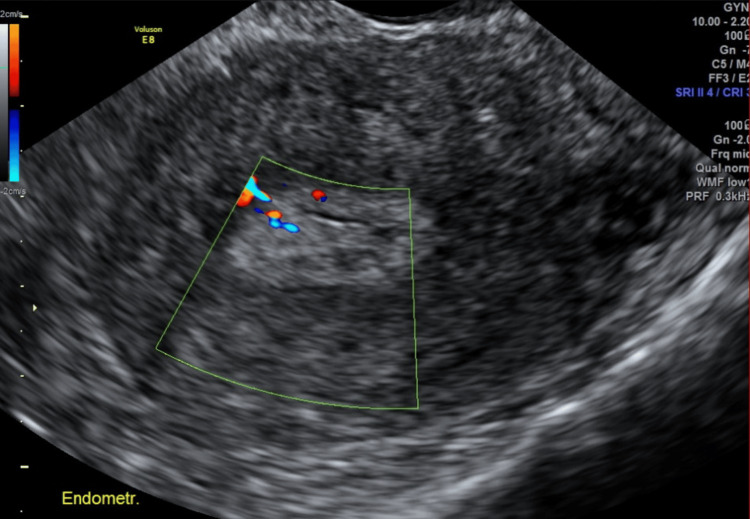
The hyperechoic oval mass observed on ultrasound with a feeding vessel is characteristic of high vascularity seen in some sarcomas.

A routine work-up was performed, and a speculum examination showed an endocervical polyp that was removed and sent for histopathology. The polyp was initially shown to be an inflamed benign endocervical polyp, but further review confirmed it as high-grade sarcoma. A pelvic ultrasound scan was performed at follow-up, which showed a slightly hyperechoic oval soft tissue mass measuring 1.2 × 0.4 cm with a feeding vessel noted on color Doppler within the endometrial cavity (Figure 1). The patient was referred for a cervical polypectomy and hysteroscopy.

**Figure 2:**
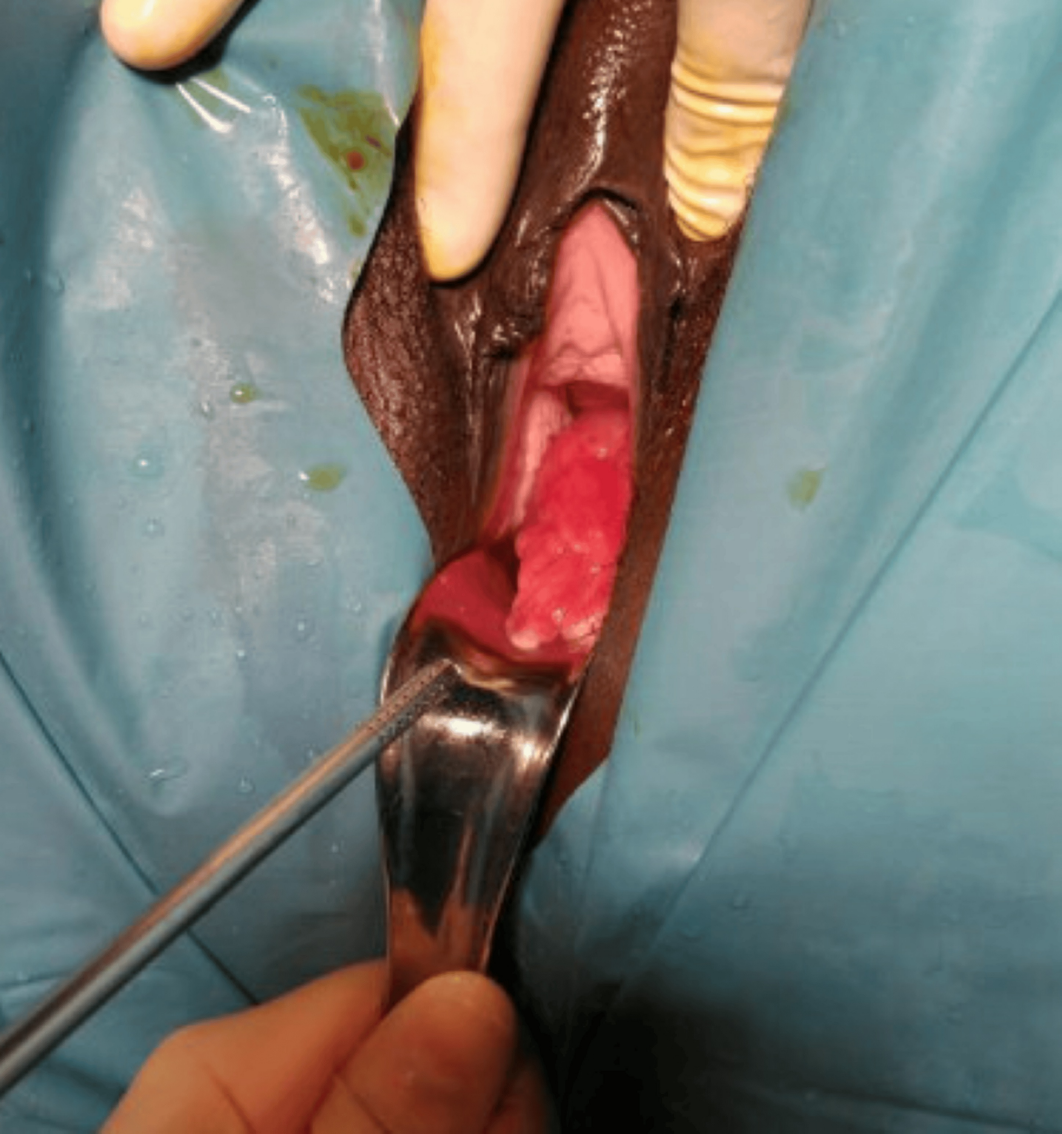
A large polypoidal mass emerging from the anterior cervical lip observed during a speculum examination.

Examination under anesthesia showed a normal vulva and vagina and a large fleshy polypoidal mass emerging from the anterior lip of the cervix. The polypoidal mass protruded until the introitus (Figure 2). The external os was open.

**Figure 3:**
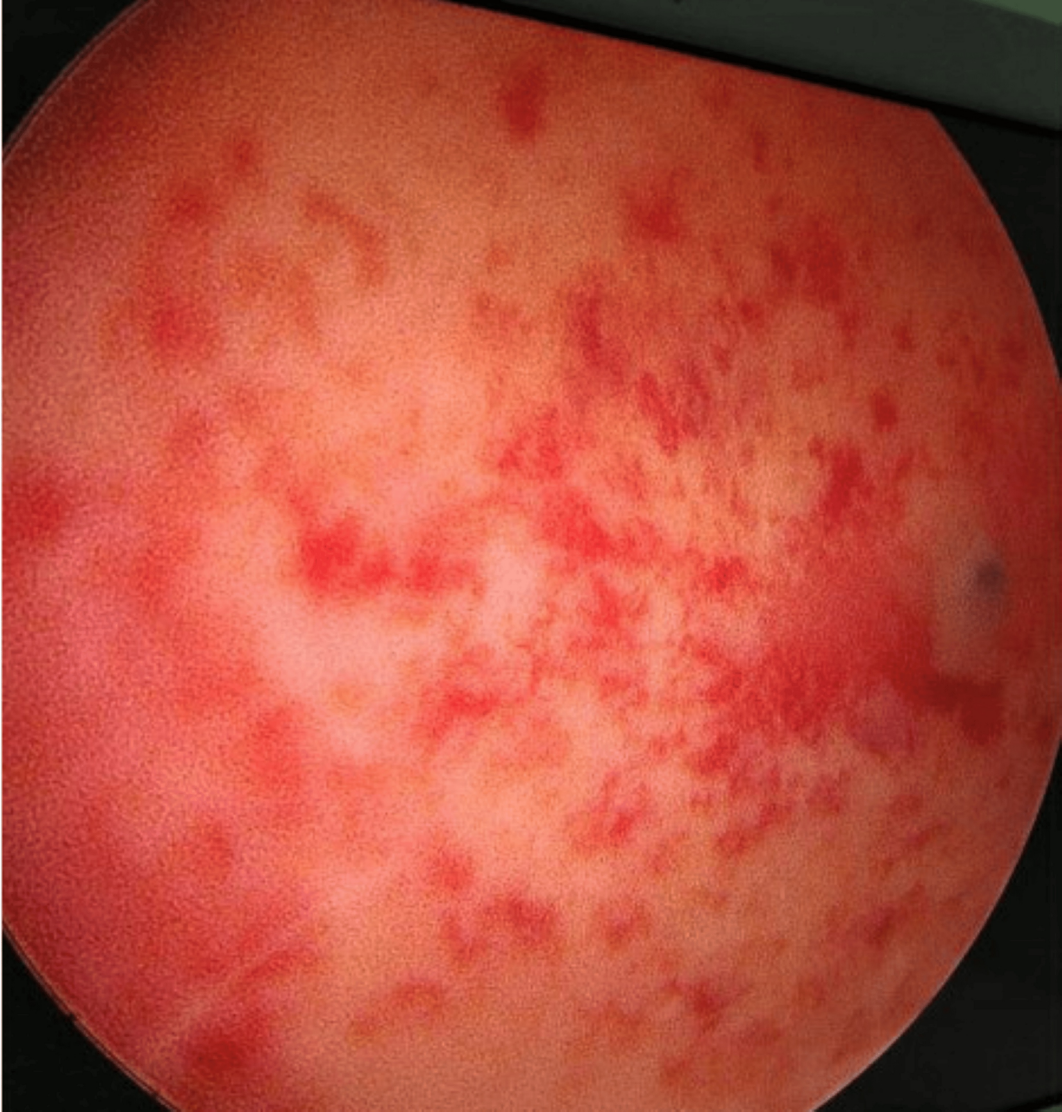
Hysteroscopic view showing a congested and vascular endometrium with no visible polyps or fibroids.

Hysteroscopy showed a normal endometrium, only congested and vascular. The uterine cavity was regular. No polyp, fibroid, or any other abnormalities were seen inside the uterine cavity or cervical canal (Figure 3).

Stay sutures were applied at 12 and 9 o’clock, and cervical polypectomy was accomplished. Following this, the base was shaved and another hemostatic stitch was placed at 2 o’clock. A specimen was sent for histopathology. The histopathology report indicated a uterine cervical/corporal embryonal rhabdomyosarcoma.

In-house immunohistochemistry (IHC) for SMA, S100, desmin, myogenin, MyoD1, calponin, caldesmon, CD10, and keratin (CKAE1/AE30 and Ki67) was performed. The neoplastic small round blue cell tumor cells were positive for desmin, myogenin, and MyoD1 with a high Ki67 proliferation index. They were negative for the remaining immunomarkers. The overall histomorphologic features and IHC results were consistent with uterine cervical/corporal embryonal rhabdomyosarcoma. Unfortunately, molecular genetic studies are not available in our laboratory and the *DICER1* gene mutation study was not done.

Computed tomography of the chest, abdomen, and pelvis was normal. A multidisciplinary tumor board meeting was held to discuss the case, where it was decided to proceed with the laparoscopic total hysterectomy with bilateral salpingo-oophorectomy, which was performed routinely. The patient recovered well postoperatively and was discharged with a follow-up planned at the gynecology clinic.

The histopathology report showed a cervix with chronic cervicitis and nabothian cyst, a small focus of residual embryonal rhabdomyosarcoma, a uterine body with disordered proliferative endometrium and one intramural leiomyoma, a right ovary with multiple follicular cysts with areas of hemorrhage and hemorrhagic corpus luteum, a left ovary with multiple follicular cysts with areas of hemorrhage and corpora albicans, and both fallopian tubes with unremarkable tubal plicae and fimbrial cysts. The final diagnosis was made of Stage 1A cervical embryonal rhabdomyosarcoma.

## Discussion

There are various classifications of rhabdomyosarcoma. The most recent World Health Organization classification in 2020 retains the four subtypes of the 2013 classification (embryonal rhabdomyosarcoma, alveolar rhabdomyosarcoma, pleomorphic rhabdomyosarcoma, and spindle cell/sclerosing rhabdomyosarcoma), with an additional mention of several novel subtypes [9].

A study on rhabdomyosarcoma in the cervix of adult and adolescent women reviewed data retrospectively over 30 years and found 11 cases, of which only five patients were above 19 years of age. All presented with vaginal bleeding or discharge, and 10 out of the 11 patients had a good prognosis, with surgery being the primary treatment in most cases [5]. A retrospective observational study by Ricciardi et al. [10] included data spanning 27 years and reported 15 patients diagnosed with adult cervical embryonal rhabdomyosarcoma [10]. This review also revealed vaginal discharge and vaginal bleeding to be the most common presenting symptoms.

Similar to our case where the IHC was positive for desmin, myogenin, and MyoD1, a case report by Jadhav et al. [7] on embryonal rhabdomyosarcoma of the cervix showed tumor cell reactivity to vimentin, desmin, MyoD1, and myogenin but non-reactivity for SMA and CK7. The Ki67 proliferative index was 50-60% in the most proliferative focus, as in our case [7]. A study by Devin et al. [6] on 94 tumors showed rare to patchy positivity in the cellular aggregates myogenin and MyoD1. Desmin was positive in all 45 tumors tested and showed more extensive staining than myogenin and MyoD1 [6]. The pathogenesis of embryonal rhabdomyosarcoma of the cervix remains unclear; however, several reports implicate germline mutations involving the DICER1 gene [7]. As molecular genetics is not available in our facility, this genetic testing was not done.

Another reported case was of a 43-year-old woman with a long-standing history of menorrhagia who was subsequently diagnosed with embryonal rhabdomyosarcoma of the cervix. She underwent a total abdominal hysterectomy, bilateral salpingo-oophorectomy, lymph node dissection, omentectomy, and appendectomy, which also revealed a synchronous tubular carcinoid tumor of appendiceal origin, and further chemotherapy was planned [2]. A similar case report by Bernal et al. [11] described an embryonal cervical rhabdomyosarcoma in a 19-year-old presenting as a cervical polyp. However, this case was managed with conservative surgery, followed by adjuvant chemotherapy, thus maintaining fertility for the young patient. Another case series of three nulliparous women, aged 14, 20, and 21 years, demonstrated fertility-sparing surgery and chemotherapy as a treatment option for selected cases of embryonal cervical rhabdomyosarcoma with good outcomes [12].

Further studies are needed to define a treatment protocol in these cases. Due to the occurrence of this malignancy during the reproductive years, fertility-sparing surgery and chemotherapy should be considered in select cases.

## Conclusions

Although rare, embryonal rhabdomyosarcoma of the cervix can present with common gynecological symptoms such as abnormal uterine bleeding. This case underscores the critical role of histopathology in achieving an accurate diagnosis. While this patient had a favorable outcome with minimally invasive surgery, the prognosis varies widely, emphasizing the need for early detection and individualized treatment strategies. Further research is required to establish standardized management protocols and explore the role of genetic and molecular profiling in improving diagnostic accuracy and therapeutic outcomes.
